# CCL5 activation of CCR5 regulates cell metabolism to enhance proliferation of breast cancer cells

**DOI:** 10.1098/rsob.160122

**Published:** 2016-06-22

**Authors:** Darrin Gao, Ramtin Rahbar, Eleanor N. Fish

**Affiliations:** 1 Toronto General Research Institute, University Health Network, Toronto, Canada; 2 Princess Margaret Cancer Centre, University Health Network, Toronto, Canada; 3 Department of Immunology, University of Toronto, Toronto, Canada

**Keywords:** breast cancer, CCL5, CCR5, glycolysis, anabolic metabolism

## Abstract

In earlier studies, we showed that CCL5 enhances proliferation and survival of MCF-7 breast cancer cells in an mTOR-dependent manner and we provided evidence that, for T cells, CCL5 activation of CCR5 results in increased glycolysis and enhanced ATP production. Increases in metabolic activity of cancer cells, specifically increased glycolytic activity and increased expression of glucose transporters, are associated with tumour progression. In this report, we provide evidence that CCL5 enhances the proliferation of human breast cancer cell lines (MDA-MB-231, MCF-7) and mouse mammary tumour cells (MMTV-PyMT), mediated by CCR5 activation. Concomitant with enhanced proliferation we show that CCL5 increases cell surface expression of the glucose transporter GLUT1, and increases glucose uptake and ATP production by these cells. Blocking CCL5-inducible glucose uptake abrogates the enhanced proliferation induced by CCL5. We provide evidence that increased glucose uptake is associated with enhanced glycolysis, as measured by extracellular acidification. Moreover, CCL5 enhances the invasive capacity of these breast cancer cells. Using metabolomics, we demonstrate that the metabolic signature of CCL5-treated primary mouse mammary tumour cells reflects increased anabolic metabolism. The implications are that CCL5–CCR5 interactions in the tumour microenvironment regulate metabolic events, specifically glycolysis, to promote tumour proliferation and invasion.

## 1. Introduction

Inflammation is critical in tumour progression [1–3], creating a tumour microenvironment largely defined by soluble secreted factors and an influx of inflammatory cells. Together, these participate in the neoplastic process to regulate the proliferation, survival and migration of tumour cells [4,5]. Notably, tumour cells have acquired some of the effectors of the innate immune system, such as chemokines and their receptors, to facilitate metastasis and invasion.

Chemokines are chemotactic cytokines responsible for orchestrating leucocyte migration. Chemokine binding to G protein-coupled receptors initiates signalling cascades that promote directional migration through cytoskeletal rearrangement, cell polarization and integrin activation. Chemokines also regulate numerous migration-unrelated responses, including cell survival, apoptosis, protein translation, embryogenesis, angiogenesis and tumour growth [6]. In the context of breast cancer, chemokines have been implicated in promoting the malignant phenotype: CXCL3, CXCL12, CXCL13, CCL21 and CCL5 are associated with tumour progression and metastasis in breast cancer [7–11].

CCL5 (RANTES) is a chemokine that exerts an important role in inflammation, by orchestrating the migration of monocytes and T cells to injured, infected and tumour sites [12]. While CCL5 may promote efficient anti-tumour immune responses, it has also been associated with cancer progression and metastasis [10,11]. Notably, CCL5 engagement with its cognate receptor, CCR5, results in the rapid upregulation of mRNA translation of pro-survival factors in MCF-7 breast cancer cells [13]. Furthermore, CCL5 is a predictor of disease progression in stage II breast cancer patients. Circulating CCL5 levels are elevated in patients with high-grade tumours compared with low-grade tumours [14].

Unlike differentiated tissue, tumour cells are more reliant on anaerobic glycolysis, even in the presence of oxygen [15]. The glycolysis pathway can generate a number of key metabolites that provide a proliferative advantage to tumour cells. Increases in metabolic activity of cancer cells, specifically increased glycolytic activity and increased expression of glucose transporters, are associated with tumour progression [16,17]. In earlier studies, we provided evidence that CCL5–CCR5 signalling can stimulate growth of MCF-7 cells in an mTOR-dependent manner [13]. In addition, for T cells, CCL5 activation of CCR5 results in increased glucose uptake and enhanced intracellular ATP, all associated with enabling CCL5-mediated chemotaxis [18].

In this report, we provide the first evidence that CCL5–CCR5 interactions regulate breast cancer cell metabolism to enhance proliferation, migration and invasion. The novelty of these data suggests targeting CCL5–CCR5 interactions that increase tumour metabolism may be an effective therapeutic strategy to limit tumour proliferation and tissue invasion.

## 2. Results

### 2.1. CCL5 increases glucose uptake, GLUT-1 expression and glycolysis

**Figure 1.**
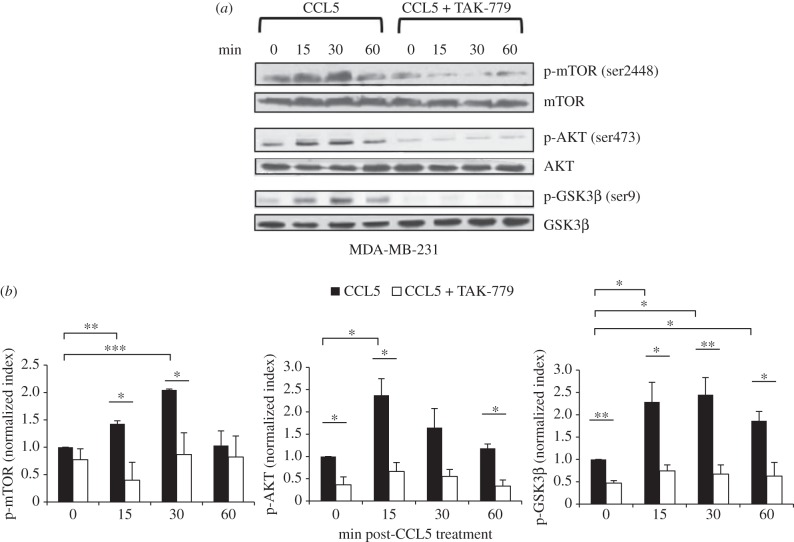
CCL5 treatment activates the mTOR/AKT pathway in breast cancer cells. (*a*) Cells were either left untreated, treated with 10 nM CCL5 for the indicated times or pre-treated with 2uM TAK-779 for 1 h prior to CCL5 treatment. Cell lysates were prepared and resolved by SDS-PAGE, and then immunoblotted with the indicated antibodies. Data shown are representative of three independent experiments. (*b*) Bands were quantified by densitometry using ImageJ software and normalized relative to the corresponding unphosphorylated proteins. Bar graphs represent means ± s.e. of the combined data from the three experiments. Statistical analysis was performed comparing different treatments: *p < 0.05, **p < 0.01 and ***p < 0.001.

In an earlier publication, we provided evidence that CCL5 treatment promotes the proliferation of MCF-7 human breast cancer cells, through mTOR-dependent mRNA translation of a subset of proteins associated with cell cycle progression and survival [13]. mRNA translation is an energy dependent process. Moreover, the TORC1 complex in the mTOR pathway functions to integrate many different signals beyond mRNA translation, including those associated with nutrient sensing. In a first series of experiments, we examined the effects of CCL5 on activation of the AKT/mTOR pathway in MDA-MB-231 human breast cancer cells. In western immunoblots of cell lysates, we identified the rapid and transient phosphorylation of AKT and mTOR following CCL5 treatment (figure 1), with evidence of phosphorylation of the downstream target of mTOR, 4E-BP1, being phosphorylated (electronic supplementary material, figure S1). In addition, we provide evidence that CCL5 phosphorylates glycogen synthase kinase 3β (GSK-3β), thereby de-repressing/releasing signalling downstream of CCR5 associated with the mTOR pathway (figure 1). Using the CCR5 inhibitor TAK-779, we show that these CCL5-mediated signalling events are mediated by CCR5 (figure 1). CCL5 activation of mTOR signalling is consistent with our earlier findings [13].

In all subsequent experiments, we employed MCF-7 and MDA-MB-231 breast cancer cells and primary cells from mammary tumours harvested from MMTV-PyMT mice. Cell surface expression of CCR5 was confirmed by fluorescence-activated cell sorting (FACS) analysis in all three breast cancer cell types (electronic supplementary material, figure S2).

**Figure 2.**
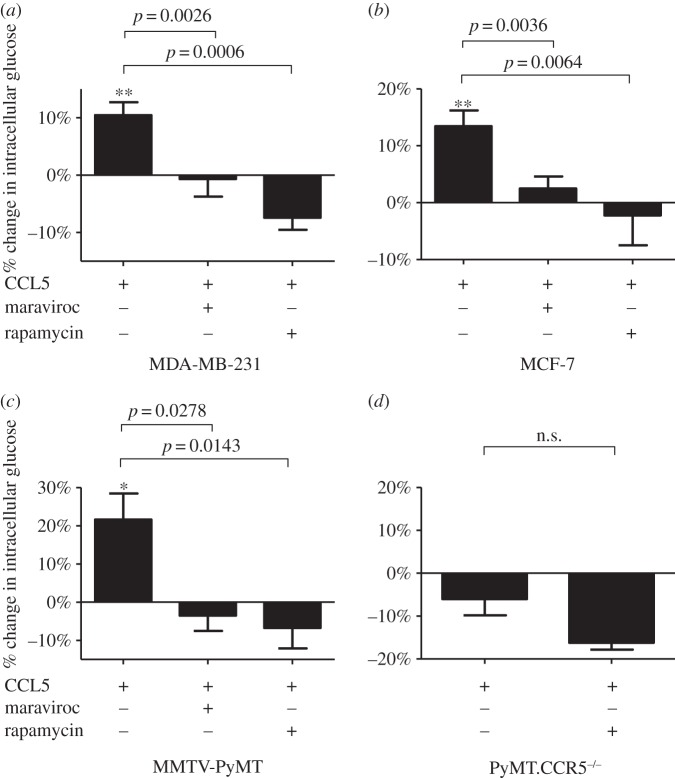
(*a*–*d*) CCL5 treatment increases glucose uptake in breast cancer cells mediated by CCR5 and mTOR. Cells were either left untreated, treated with 10 nM CCL5 for 3 h or pre-treated with 2 µM maraviroc or 50 nM rapamycin for 1 h prior to CCL5 treatment. Glucose uptake was measured at 3 h, using 2-NDBG, as described in Material and methods. Glucose uptake is expressed as percentage change relative to untreated cells. Values are means ± s.e. of triplicate assays and each data point combines the data from three independent experiments. Statistical analysis was performed comparing different treatments, with *p*-values as indicated, or comparing CCL5 treatment with untreated cells: *p < 0.05 and **p < 0.01.

In T cells, we have shown that CCL5 regulates glucose uptake and ATP generation, required to enable their migration [18]. Notably, CCL5 regulation of glucose uptake was mTOR-dependent. Having shown that T cell chemotaxis is facilitated by CCL5 regulating glucose uptake [18], we speculated that CCL5 may enhance tumour cell proliferation, invasion and metastasis by a similar mechanism, because cancer cells exhibit significant dependence on glucose for their growth and survival [19]. At the outset, we examined the effects of CCL5 treatment of the different breast cancer cell types on glucose uptake. For these experiments, we employed the fluorescent glucose analogue 2-NBDG. The results in figure 2*a–c* show that at a physiologic dose of 10 nM, CCL5 increases glucose uptake for all three breast cancer cell lines and that this uptake is CCR5- and mTOR-dependent, as both maraviroc (CCR5 inhibitor) and rapamycin (mTOR inhibitor) inhibit uptake. Our time course studies revealed that glucose uptake is elevated 1 h after CCL5 treatment, reaching a maximum by 3 h and remaining elevated for 18 h (electronic supplementary material, figure S4). These findings were similar whether 10 or 50 nM of CCL5 was administered (data not shown). Notably, when cells derived from tumours harvested from MMTV-PyMT.CCR5^−/−^ mice (described in Material and methods) are treated with CCL5, we observe no enhancement in glucose uptake, in support that CCR5 is the receptor mediating this effect (figure 2*d*).

**Figure 3.**
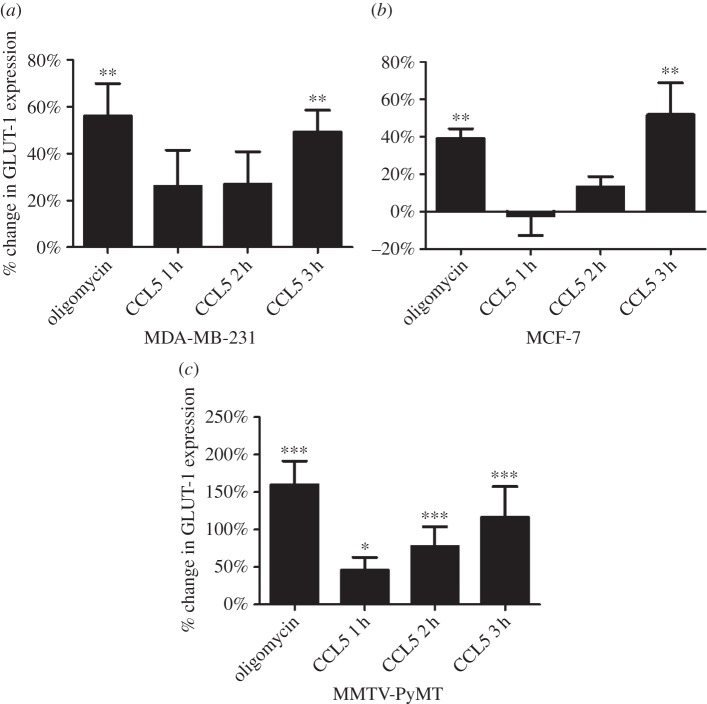
(*a*–*c*) CCL5 treatment increases GLUT-1 expression. Cells were treated with 10 nM CCL5 for 1, 2 and 3 h, or 2 µM oligomycin for 3 h, then fixed with 2% formalin prior to staining for cell surface GLUT-1 expression and FACS analysis. GLUT-1 expression is expressed as percentage change relative to untreated cells. Values are means ± s.e. of triplicate assays and each data point combines the data from three independent experiments. Statistical analysis was performed comparing untreated and CCL5/oligomycin treated samples: *p < 0.05, **p < 0.01 and ***p < 0.001.

Glucose transporter (GLUT) proteins facilitate glucose uptake across the plasma membrane. In human cells, 14 different GLUT isoforms have been identified, of which GLUT-1, GLUT-3 and GLUT-12 have been reported in clinical breast cancer cells (reviewed in [20]). Given that GLUT-1 expression is upregulated by AKT activation [21] and we have shown CCL5 activation of AKT, we examined the effects of CCL5 treatment on GLUT-1 cell surface expression for the different breast cancer cells. Oligomycin (inhibitor of mitochondrial oxidative respiration) treatment, that diverts glucose preferentially for glycolysis, served as the positive control. The results in figure 3 show that CCL5 treatment increases cell surface GLUT-1 expression over 3 h for all three breast cancer cell types. CCL5 treatment had no effect on GLUT-2, GLUT-3 or GLUT-4 cell surface expression (data not shown).

**Figure 4.**
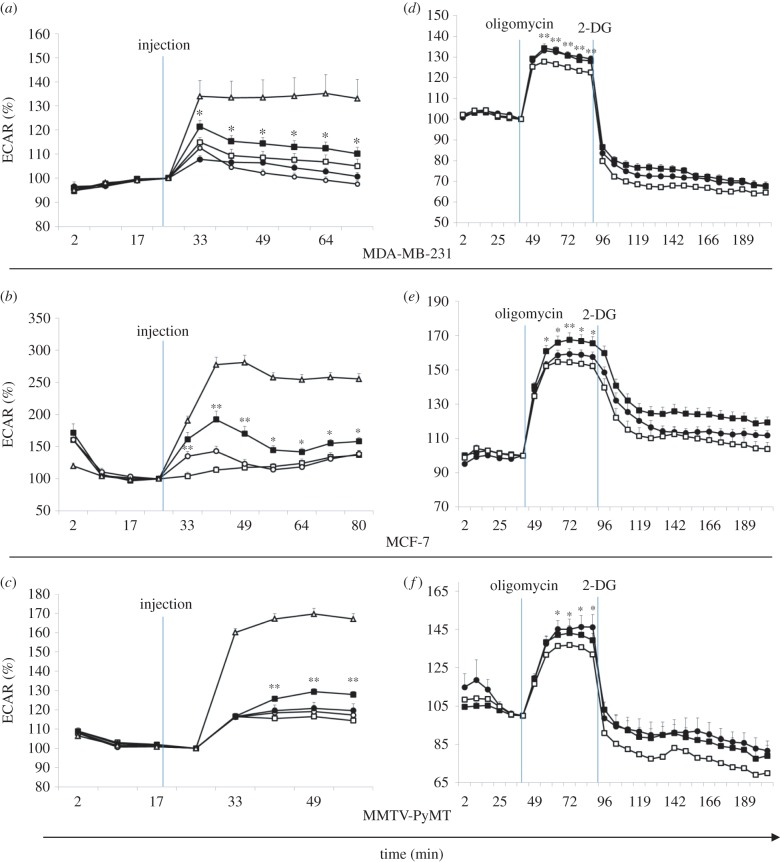
CCL5 increases rate of glycolysis and cellular glycolytic capacity. A Seahorse Extracellular Flux Analyzer was used to detect real-time changes in extracellular acidification rate (ECAR) as measurements of the rate of glycolysis. (*a–c*) The indicated compounds were injected at the indicated time. Percentage changes in ECAR were measured, □, medium; ▪, CCL5; ○, CCL5 + maraviroc; •, CCL5 + rapamycin; Δ, oligomycin. (*d–f*) Glycolytic stress test. Cells were untreated (medium alone) or treated with 10 nM CCL5 for 3 or 23 h. As indicated 2 µM oligomycin was introduced followed by the glycolysis inhibitor 2-DG, at the indicated times. Percentage change in ECAR was measured. □, medium; ▪, 10 nM CCL5, 3 h; •, 10 nM CCL5, 23 h. Values are the means ± s.e. of three combined independent experiments, with all treatments being performed as 12 replicates. Statistical analysis was performed comparing no treatment with CCL5 treatment: *p < 0.05 and **p < 0.01.

Glucose uptake drives anaerobic glycolysis and ATP production. Most cancers rely on glycolysis for ATP production despite an available oxygen source. Glycolytic production of lactate serves as a measure of metabolic activity. Accordingly, we undertook a series of time course experiments using the Seahorse Metabolic Flux analyzer to evaluate the effects of CCL5 on glycolytic flux, using extracellular acidification rate (ECAR) as the readout. The data in figure 4*a–c* represent the time course of ECAR measurements when cells are treated with CCL5. Although CCL5 treatment increased ECAR, pre-treatment with rapamycin or maraviroc ablated this increase in all three breast cancer cell types. Oligomycin treatment served again as the positive control for glycolysis. To investigate the effect of CCL5 on cellular capacity to conduct glycolysis, cells were pre-treated with CCL5 for 3 or 23 h, at which time oligomycin was introduced to preferentially force glycolysis. The difference between the basal level of ECAR and the maximal ECAR reached in the presence of oligomycin is the glycolytic reserve/capacity of the cell. Cells that were treated with CCL5 exhibit an elevated glycolytic reserve compared with untreated cells (figure 4*d–f*). 2-Deoxy-d-glucose (2-DG), an inhibitor of glycolysis, was subsequently added, validating that the observed changes in ECAR were due to changes in the glycolytic rate.

**Figure 5.**
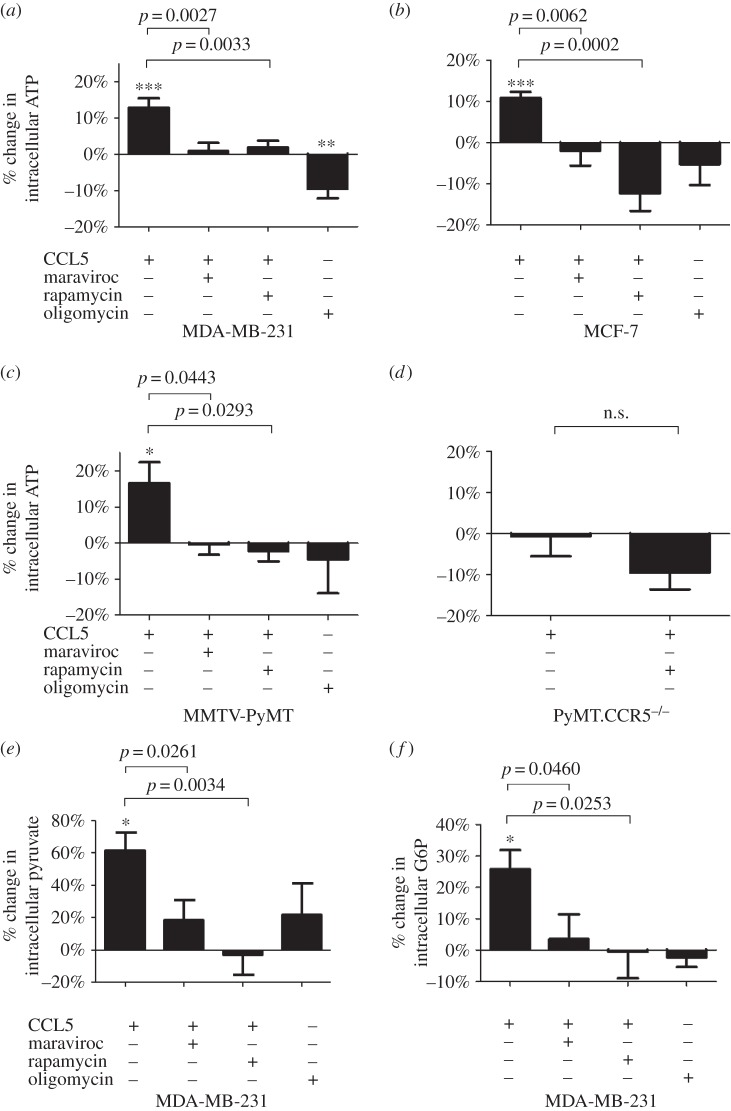
CCL5 treatment increases ATP and glycolytic intermediate flux. (*a–d*) Cells were either left untreated, treated with 10 nM CCL5 for 3 h or pre-treated with 2 µM maraviroc or 50 nM rapamycin for 1 h prior to CCL5 treatment for 3 h. Oligomycin is included as a control. The percentage change in intracellular ATP levels relative to untreated (medium alone) cells is shown. Values are means ± s.e. of triplicate assays and each data point combines the data from two independent experiments. Statistical analysis was performed comparing different treatments, with *p*-values as indicated or comparing untreated cells with CCL5 treatment: *p < 0.05, **p < 0.01, ***p < 0.001. MDA-MB-231 cells were either left untreated, treated with 10 nM CCL5 for 3 h or pre-treated with 2 µM maraviroc or 10 nM rapamycin for 1 h prior to CCL5 treatment for 3 h. The percentage change in intracellular pyruvate (*e*) and glucose 6-phosphate (G6P) (*f*) relative to untreated (medium alone) cells is shown. Values are means ± s.e. of triplicate assays and each data point combines the data from two independent experiments. Statistical analysis was performed comparing different treatments, with *p*-values as indicated or comparing untreated cells with CCL5 treatment: *p < 0.05.

In support of the effects of CCL5 treatment on enhancing glycolysis, we observed a concomitant increase in intracellular ATP levels following CCL5 treatment, sensitive to maraviroc and rapamycin treatments (figure 5*a–c*). As for glucose uptake, treatment of tumour cells from MMTV-PyMT.CCR5^−/−^ mice with CCL5 did not cause an increase in ATP production (data not shown). Moreover, CCL5 treatment increased levels of glucose 6-phosphate (G6P) and pyruvate, sensitive to treatment with maraviroc and rapamycin (figure 5*d–e*). For G6P, the first intermediate in glycolysis, levels were significantly higher than for pyruvate, the final intermediate, suggesting that the majority of the glycolytic intermediates are shuttled into other, potentially anabolic, pathways.

### 2.2. Metabolic signature of CCL5-treated cancer cells reflects increased anabolic metabolism

**Figure 6.**
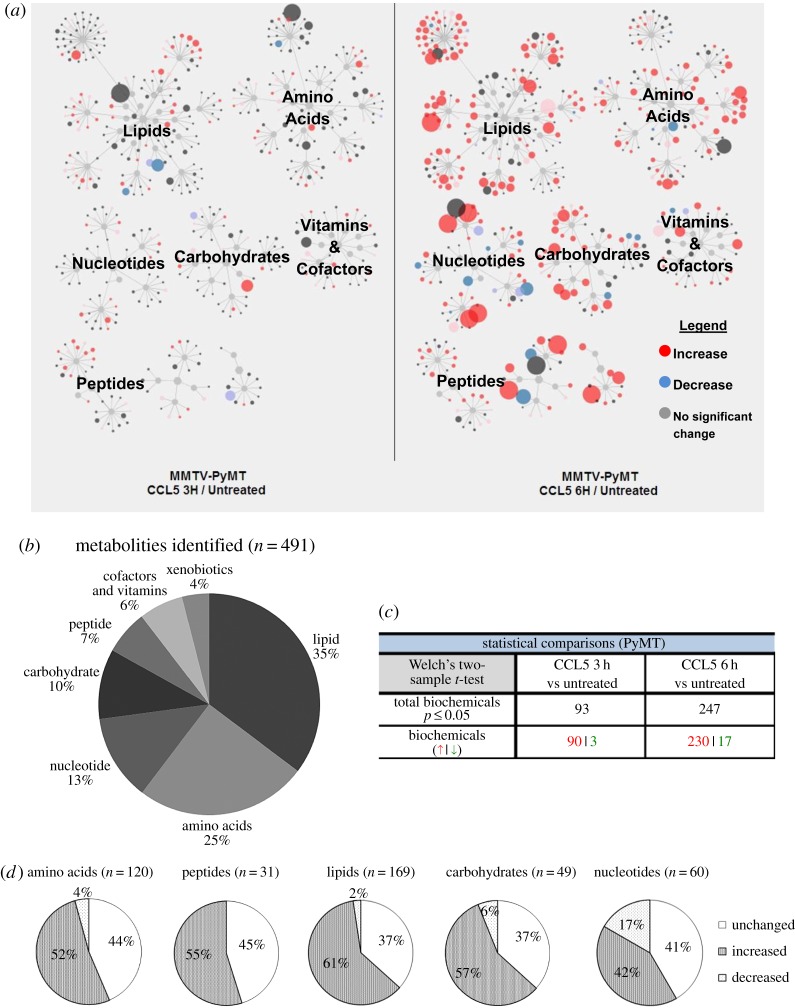
Metabolic signature of CCL5-treated MMTV-PyMT primary mouse breast cancer cells reflects increased anabolic metabolism. MMTV-PyMT cells were treated with medium alone or 10 nM CCL5 for 3 or 6 h (*n* = 4 for each treatment). Cells were pelleted by centrifugation and stored frozen for transfer to Metabolon. (*a*) Schematic diagram illustrating the fold-change of metabolites in different metabolic pathways in MMTV-PyMT mouse breast cancer cells after 3 and 6 h of CCL5 treatment. The size of the circle indicates the degree of change. The colour indicates the direction of the change in flux, as indicated. (*b*) In total, 491 metabolites were identified, grouped according to their classification and as a percentage of the total number identified. (*c*) Welch 2-sample *t*-tests were used to identify metabolites whose levels changed significantly upon CCL5 treatment at the indicated times, relative to their levels in untreated cells (*p* < 0.05). Red indicates an increase in expression and green, a decrease. (*d*) Pie chart distinguishing changes in levels of metabolites following 6 h of CCL5 treatment.

**Figure 7.**
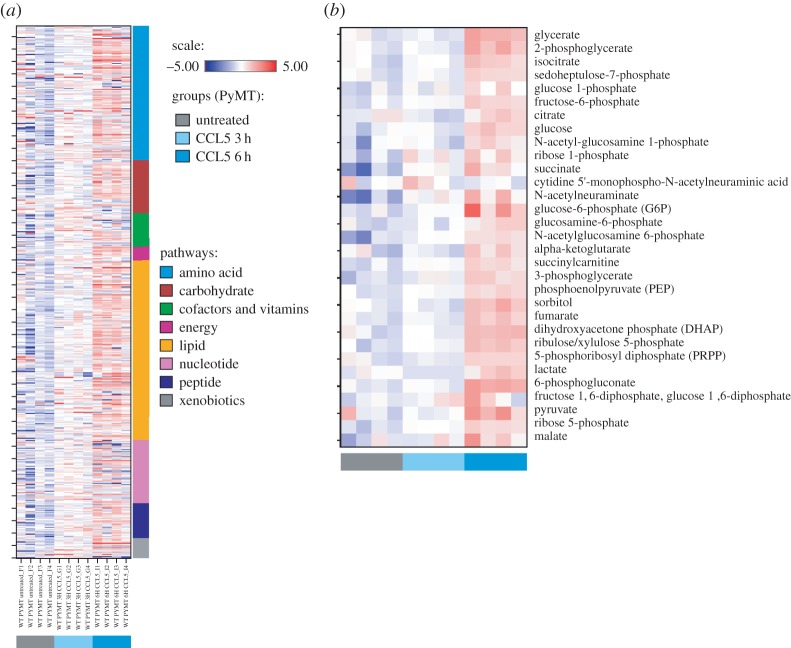
Heat maps reveal a metabolic signature in CCL5-treated MMTV-PyMT primary mouse breast cancer cells. (*a*) Heat map showing hierarchical clustering of metabolic flux in MMTV-PyMT cells treated with CCL5 for 3 and 6 h (four replicates per each treatment group for a total of 12 samples). The *Y*-axis is clustered according to metabolic pathways. (*b*) Plotted in the heat map is a hierarchical clustering of glycolytic metabolites affected by CCL5 treatment.

Global unbiased metabolomic analysis was conducted to identify any changes in levels of metabolites that occurred following CCL5 treatment of the primary MMTV-PyMT tumour cells for 3 and 6 h (figure 6*a*). This approach identified a total of 491 metabolites. The distribution of metabolites affected by CCL5 treatment according to specific metabolic pathways is shown, with a significant proportion involved in lipid, amino acid and carbohydrate metabolism (figure 6*b*). Closer analysis revealed that CCL5 treatment for 3 h altered the levels of 93 metabolites, increasing 90 and decreasing 3, whereas CCL5 treatment for 6 h increased the levels of 230 and decreased the levels of 17 (figure 6*c*). Comparing the metabolome of cells treated with CCL5 for 6 h to untreated, nearly half of the metabolites detected in peptide, carbohydrate, lipid, amino acid and nucleotide pathways have increased, providing evidence for a global increase in cellular metabolism (figure 6*d*). Heat maps of the 491 metabolites arranged by their metabolic pathways are shown in figure 7*a*, and by glycolytic metabolites, in figure 7*b* (refer also to electronic supplementary material, table S1 for quantitation).

**Figure 8.**
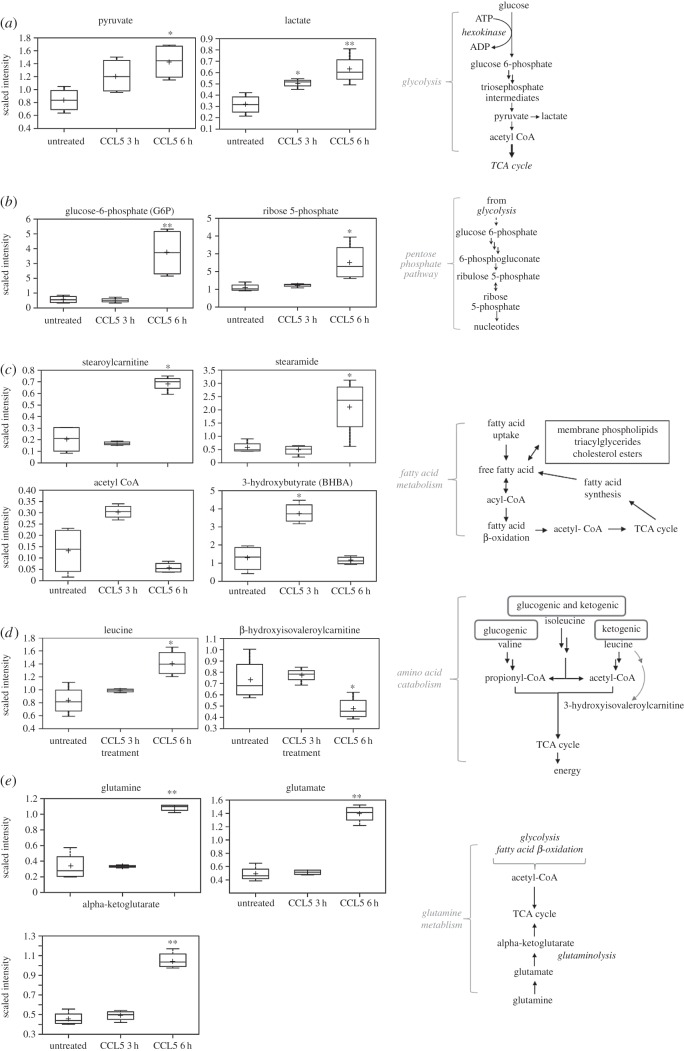
CCL5 treatment results in accumulation of metabolites. Box plots of metabolites affected by CCL5 treatment involved in (*a*) glycolysis, (*b*) pentose phosphate pathway, (*c*) fatty acid β-oxidation, (*d*) amino acid catabolism and (*e*) glutamine metabolism are identified. Box legend: + inside box represents mean value, bar inside box represents median value, upper bar represents maximum of distribution and lower bar represents minimum of distribution. Scaled intensity is the normalized concentration of the metabolite relative to the Bradford protein concentration. Statistical analysis was performed comparing untreated and CCL5-treated cells: *p < 0.05, **p < 0.01. Schematics of the distinct pathways are indicated.

Consistent with our *in vitro* findings, closer scrutiny of this metabolomic analysis revealed an increase in metabolites involved in glucose metabolism (figure 8*a*). Following cellular uptake, glucose is phosphorylated to G6P, which can be metabolized either by glycolysis or by the pentose phosphate pathway. By 6 h after CCL5 treatment, the glycolytic intermediates pyruvate and lactate were elevated (figure 8*a*). Similarly, specific metabolites of the pentose phosphate pathways, namely G6P and ribose 5-phosphate levels, were elevated after CCL5 treatment (figure 8*b*).

Fatty acids are a critical source of energy for mitochondrial oxidation and cellular ATP generation. Fatty acids are broken down into acetyl CoA, which can then be directed into the tricarboxylic acid (TCA) cycle for ATP production. When in excess, acetyl CoA can be synthesized into ketone bodies, namely 3-hydroxybutyrate (BHBA) during ketogenesis. CCL5 treatment resulted in a transient increase in β-oxidation at 3 h, as reflected in the modest decrease in the levels of fatty acids, including stearoylcarnitine, stearamide, as well as a significant increase in the level of acetyl CoA and its downstream product BHBA (figure 8*c*). By 6 h after CCL5 treatment, these changes were reversed.

CCL5 treatment increased the accumulation of branched-chain amino acids (BCAAs), including leucine (figure 8*d*). As shown, CCL5 induces a steady and consistent increase in the concentrations of BCAAs, while the level of β-hydroxyisovalerylcarnitine, an intermediate for BCAA catabolism, is decreased over the course of the treatment (figure 8*d*). In addition, consistent with other anabolic pathways examined, the levels of glutamine as well as its downstream intermediates, including glutamate and alpha-ketoglutarate, increased post-CCL5 treatment (figure 8*e*; electronic supplementary material, table S1).

### 2.3. CCL5 increases the proliferation, migration and invasive capacity of breast cancer cells

**Figure 9.**
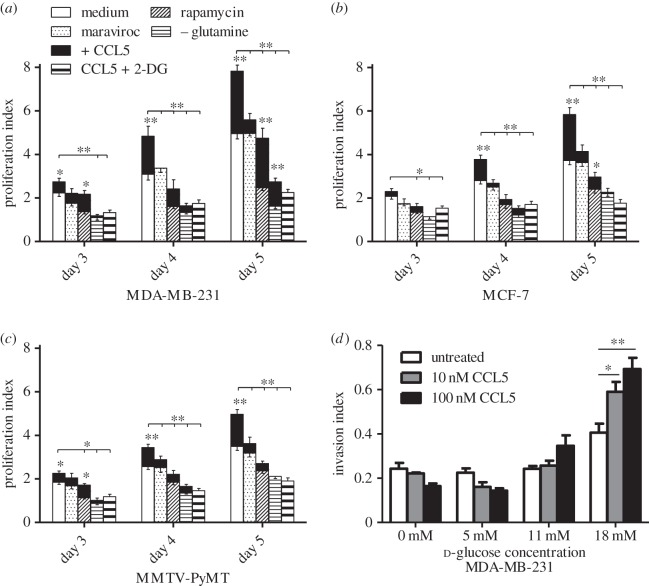
CCL5 treatment enhances cell proliferation and invasion. (*a–c*) Cells were either left untreated (medium alone), treated with 2 µM maraviroc (control), 50 nM rapamycin (control) or 10 nM CCL5 or pre-treated with the aforementioned inhibitors for 1 h prior to CCL5 treatment. In addition, cells were pre-treated with 2 mM 2-DG for 1 h prior to CCL5 treatment. In contrast with all the previous treatments in medium containing 5 mM glutamine, an additional time course was conducted with 10 nM CCL5 treatment, in medium that contained no glutamine (CCL5 –glutamine). For all, medium was changed every other day and the treatment(s) reapplied. At the indicated time points, cells were trypsinized and counted with a haemocytometer. The profliferation index is normalized against untreated input cell number (i.e. cells at time zero). Values are means ± s.e. of triplicate assays and each data point combines the data from three independent experiments. Statistical analysis was performed comparing untreated cells with CCL5-treated cells and inhibitor-treated cells with CCL5 + inhibitor-treated cells, or comparing CCL5-treated with CCL5 + inhibitor-treated cells: *p < 0.05, **p < 0.01. (*d*) In total, 10^5^ cells in 100 µl buffer containing CCL5 at the indicated dose were placed in the upper compartment of the invasion chambers. Cells were allowed 24 h to invade into the bottom compartment through the ECMatrix. Cells were subsequently quantified using CyQuant GR DyeCells. Values are means ± s.e. of triplicate assays and each data point combines the data from two independent experiments. Statistical analysis was performed comparing different treatments, with *p*-values as indicated: *p < 0.05, **p < 0.01.

Consistent with our earlier data, in a 5 day time course study, we show that CCL5 treatment increases the proliferation of the three breast cancer cell lines. (figure 9*a–c*). As anticipated, MMTV-PyMT.CCR5^−/−^ cells show no increase in proliferation/normal growth when treated with CCL5 (data not shown)*.* Although maraviroc treatment alone has no effect on the proliferation of each of the cell lines (within s.e. of proliferation index), as anticipated, rapamycin treatment reduced their proliferation over the 5-day time period. When cells are pre-treated with maraviroc or rapamycin, then CCL5 added, the proliferative response to CCL5 is significantly reduced over the 5-day time course. Cells grown in glutamine-free medium exhibit a significantly blunted proliferation compared with their growth in medium with glutamine. When treated with CCL5, however, cell proliferation is enhanced. In a final series of treatments in these proliferation studies, we show that CCL5 treatment does not enhance cell proliferation when glucose uptake is blocked using 2-DG.

Next, we examined the effects of CCL5 treatment on the migration/chemotaxis and invasive capacity of the different breast cancer cell types. We confirmed our earlier findings in MCF-7 cells that CCL5 promotes cell migration in a CCR5- and mTOR-dependent manner, using MDA-MB-231 cells (electronic supplementary material, figure S3). We provide evidence that CCL5-mediated cell invasion is sensitive to the nutrient supply in the extracellular environment: although CCL5 treatment induced an approximately twofold increase in invasion under standard cell culture glucose concentrations (18 mM), at lower glucose concentrations (12 mM, 6 mM or 0 mM), CCL5 treatment did not increase invasion (figure 9*d*).

## 3. Discussion

Breast cancer is the most common cancer and the most common cause of cancer-related death among women. CCL5 and CCR5 are overexpressed in basal and HER-2+ breast cancer subtypes, but not in normal breast epithelial cells [22,23]. Notably, the *CCL5* gene is located in close proximity to *Her2* on chromosome 17, probably contributing to their associated expression patterns. Accumulating data indicate that the CCL5–CCR5 axis is associated with promoting breast cancer proliferation, invasion and metastasis. Herein, we provide the first evidence of CCL5-inducible signalling events in breast cancer cells, mediated by CCR5, that regulate metabolic activity to support enhanced cell proliferation, migration and invasion facilitating tumour progression.

We demonstrate CCL5-inducible rapid activation of the mTOR/AKT pathway as well as phosphorylation of a downstream substrate, GSK3β, in breast cancer cells. mTOR and AKT are central sensors of nutrients, regulating the biosynthesis of proteins, nucleotides and lipids and inhibiting catabolic processes such as autophagy [24]. The mTOR/AKT pathway is associated with the transcriptional induction of genes that encode critical enzymes for the pentose phosphate pathway and for *de novo* pyrimidine synthesis and genes associated with lipogenesis. Moreover, mTOR-dependent regulation of glucose uptake and GLUT-1 expression has been reported [25]. In agreement, we demonstrate that CCL5 treatment increases glucose uptake by breast cancer cells, mediated by CCR5 and mTOR-dependent. We observe a concomitant increase in GLUT-1 cell surface expression. AKT regulation of glycolysis includes localization of GLUT-1 to the cell surface and regulation of hexokinase activity, required for phosphorylation of G6P [26]. We provide evidence that CCL5 treatment increases G6P and pyruvate levels, also mediated by CCR5 and sensitive to rapamycin. We provide evidence that elevated levels of G6P were evident at 6 h after CCL5 treatment, but not after 3 h, yet the levels of subsequent downstream intermediates were elevated at both the 3 and 6 h time points. This is probably due to the increased demand for G6P at 3 h following CCL5 treatment, as a substrate for both glycolysis and the pentose phosphate pathway, while hexokinase activity, which is associated with replenishing the G6P supply by catalysing the phosphorylation of glucose, may not be sufficiently elevated to support this increase in G6P demand. By 6 h after CCL5 treatment, the rate of G6P production may be sufficient to exceed that of G6P consumption, resulting in a significant increase in G6P flux. Furthermore, hexokinase activity is self-limiting and therefore depends on the concentration of G6P. Cells may temporarily limit the level of G6P in order to maximize the catalytic activity of hexokinase, allowing for sustained and elevated glycolytic flux.

Our metabolomic analysis revealed CCL5-induced increases in both pyruvate and lactate levels. The relative levels of pyruvate and lactate are maintained at steady state equilibrium. Lactate is produced from pyruvate in a reaction catalysed by lactate dehydrogenase. The reaction is so rapid that pyruvate and lactate can be considered to always be in equilibrium. Our data support this (electronic supplementary material, table S1). We found that CCL5 treatment increased extracellular acidification, a readout for glycolytic flux, and also increased the glycolytic capacity of breast cancer cells. Our experiments demonstrated that CCL5-inducible increases in glucose uptake and elevated glycolytic flux led to ATP production.

Using the Metabolon platform, we further interrogated the CCL5-inducible changes in metabolism and identified increases in the levels of intermediates in multiple anabolic pathways, including glycolysis, the pentose phosphate pathway, amino acid synthesis and lipid metabolism. This accumulation of metabolites suggests the potential for diversion of intermediates into alternate pathways for macromolecule synthesis, a metabolic signature consistent with requirements for cell proliferation and invasion. Indeed, we provide evidence that CCL5 treatment enhances the proliferation of breast cancer cells and promotes their migration and invasion. Moreover, the accumulation of the BCAAs valine, isoleucine and leucine and their metabolites allows for their integration into proteins and/or processing to meet energy demands. Likewise, the accumulation of glutamine, glutamate and alpha-ketoglutarate suggests that CCL5 may also influence glutamine catabolism as an alternative source of carbon for biosynthetic pathways. Glucose and glutamine are the primary carbon sources for ATP production and biosynthesis in proliferating cells. This requirement for glutamine is particularly relevant in tumour cells, when glutamine donates its nitrogen and carbon for different growth-promoting pathways and may often become conditionally essential. The data in figure 9 show that the growth of the three breast cancer cell lines is severely limited in glutamine-deficient medium, yet addition of CCL5 enhances their growth. The metabolomics data provided evidence for CCL5 increasing levels of glutamine, from which we infer that CCL5-induced production of glutamine facilitates the enhanced proliferation observed in glutamine-deficient medium. In the tumour microenvironment, where breast cancer cells interact with adipocytes, the fibrous stroma and the immune cells of the inflammatory infiltrate, CCL5 production increases and is sustained [22], thereby ensuring that CCL5 activation of CCR5 will provide a steady supply of glutamine to support the aggressive proliferation of breast cancer cells. Notably, there is evidence that adipocytes secrete higher amounts of CCL5 in the presence of glucose and fatty acids [23], suggesting autocrine regulation of CCL5 in the tumour microenvironment.

Acetyl CoA is produced during glycolysis and from fatty acid degradation (β-oxidation) and shuttles into the TCA cycle to generate energy. CCL5 treatment increases glycolysis and at 3 h we found a corresponding spike in acetyl CoA levels that coincided with a reduction in stearoylcarnitine and several fatty acid derivatives and an increase in β-hydroxybutyrate, which is synthesized from acetyl CoA. By 6 h, acetyl CoA and β-hydroxybutyrate levels are reduced, probably reflecting the increased demand for acetyl CoA for lipogenesis.

Viewed altogether, the data indicate that the effects of CCL5 treatment on the different breast cancer cell types are associated with enhanced metabolic activity that would support the energy and biosynthetic demands of tumour cell proliferation, migration and invasion. We have evidence of CCL5 invoking increases in cell proliferation and migration, mediated by CCR5, and that CCL5 promotes invasion, dependent on glucose. Certainly, published reports of a role for CCR5 in increased breast tumour metastasis and invasion [27,28] align with these findings of CCL5 activating CCR5 to invoke a cascade of signalling pathways increasing the metabolic capacity of cells to enable these events. Further consideration of the role of the alternate CCR5 ligands, CCL3 and CCL4, on metabolic events is warranted. It is intriguing to speculate that our studies with CCL5 and CCR5 may have broader implications for other chemokine/chemokine receptor interactions purported to be important in neoplasias. A recent publication implicates CCR6 in promoting breast cancer initiation and progression [29]. In the absence of consideration of metabolic regulation, their data suggest that CCR6 influences pro-tumourigenic tumour-associated macrophage (TAM) recruitment to the tumour microenvironment [29]. Our ongoing studies are directed towards dissecting the different contributions of CCL5–CCR5 interactions in the breast tumour microenvironment, in the context of understanding how metabolic regulation may also influence the immunophenotype–TAM recruitment—and tumour onset and progression.

## 4. Material and methods

### 4.1. Mice

C57BL/6 MMTV-PyMT mice were provided by P. Ohashi (University Health Network, Toronto). The MMTV-PyMT transgene (MMTV-PyMT) has the mouse mammary tumour virus (MMTV) long terminal repeat upstream of a cDNA sequence encoding the polyoma virus middle T antigen (PyMT). Female mice develop palpable tumours by five weeks of age. C57BL/6 CCR5^−/−^ mice were purchased from the Jackson Laboratory. C57BL/6 MMTV-PyMT.CCR5^−/−^ were generated by breeding MMTV-PyMT.CCR5^+/+^ mice with C57Bl/6 CCR5^−/−^ mice, selecting for PyMT.CCR5^+/−^ F1 progeny, and then crossing PyMT.CCR5^+/−^ with C57BL/6 CCR5^−/−^ mice and selecting for PyMT.CCR5^−/−^ F2 progeny. Multifocal tumours (800–1500 mm^3^) were harvested from MMTV-PyMT and MMTV-PyMT.CCR5^−/−^ mice, tissue minced, then digested with digestion buffer (F-12, 100 units ml^−1^ penicillin, 100 mg ml^−1^ streptomycin, 1 mg ml^−1^ DNase, 100 U ml^−1^ Collagenase type I). The resultant cell suspension was passed through a 40 µm filter to obtain single cell suspension. The single cell suspensions were maintained in F12 medium supplemented with 10% fetal calf serum (Sigma), 100 units ml^−1^ penicillin and 100 mg ml^−1^ streptomycin (Invitrogen).

### 4.2. Cells and reagents

Human breast cancer cell lines MDA-MB-231 and MCF-7 were a gift from L. Penn (University Health Network, Toronto). Cell lines were maintained in DMEM/F12 medium supplemented with 10% fetal calf serum (Sigma), 100 units ml^−1^ penicillin and 100 mg ml^−1^ streptomycin (Invitrogen). CCR5 expression was confirmed by flow cytometry using an anti-human (BD BioSciences) and anti-mouse (BioLegend) CCR5 (CD195) antibody. Antibodies for phospho-mTOR (Ser-2448; #2971), mTOR (#2972), phospho-AKT (Ser-473; #9271), AKT (#9272), phospho-GSK3β (Ser-9; #9336), GSK3β (#9315) and phospho-4E-BP1 (#2855) were purchased from Cell Signaling Technology. GLUT-1, -2, -3 and -4 antibodies were obtained from Santa Cruz Biotechnology Inc. and R & D Systems. CCL5 was a generous gift from A. Proudfoot (Geneva Research Centre, Merck Serono International, Switzerland). Maraviroc and TAK-779 (*N*,*N*-dimethyl-*N*-([[2-(4-methylphenyl)-6,7-dihydro-5*H*-benzocyclohepten-8-yl]amino]benzyl)-tetrahydro-2*H*-pyran-4-aminium chloride) were provided by D. Branch (University of Toronto). Rapamycin was obtained from Calbiochem. 2-DG, the ATP bioluminescent assay, pyruvate assay and glucose-6-phosphate assay kits were purchased from Sigma. Oligomycin was obtained from Cell Signaling Technology.

### 4.3. Immunoblotting and immunoprecipitation

Cells were serum starved for 16 h, then incubated with 10 nM CCL5 for the indicated times, pelleted by centrifugation, then lysed in 100 µl of lysis buffer (1% Triton X-100, 0.5% NP-40, 150 mM NaCl, 10 mM Tris-HCl, pH 7.4, 1 mM EDTA, 1 mM EGTA, 0.2 mM PMSF). In total, 40 µg of protein lysate was denatured and resolved by SDS-PAGE. The separated proteins were transferred to a nitrocellulose membrane and membranes were probed with the specified antibodies. Proteins were visualized using the ECL detection system.

### 4.4. Fluorescence-activated cell sorting analysis

In total, 10^6^ cells were incubated with the specified primary antibodies or isotype control antibodies, for 30 min at room temperature, followed by 20 min with FITC/PE-conjugated secondary antibodies, then stained and fixed immediately in 2% PFA. Cells were analysed using the FACSCalibur and FlowJo software (BD Biosciences).

### 4.5. Glucose uptake assay

In total, 3 × 10^5^ cells in 2% FCS DMEM/F12 medium were plated in individual wells of 12-well plates overnight. Cells were either left untreated, pre-treated with inhibitors for 1 h prior to CCL5 treatment or treated with CCL5 alone for the indicated times, then pulsed with 50 µM 2-deoxy2-[(7-nitro-2, 1,3-benzoxadiazol-4-yl) amino]-d-glucose (2-NBDG) for 15 min. Cells were washed three times with PBS, detached using 1 mM EDTA and analysed using the FACSCalibur and FlowJo software (BD Biosciences).

### 4.6. ATP bioluminescent assay

Intracellular ATP levels were examined using the ATP bioluminescent assay kit, according to the manufacturer's protocol (Sigma-Aldrich Co. LLC). Briefly, 2 × 10^4^ cells were either left untreated, pre-treated with inhibitors for 1 h prior to treatment with CCL5 or treated with CCL5 alone. Bioluminescence was measured using a VICTOR™ X3 Multilabel Plate Reader (PerkinElmer).

### 4.7. Glucose 6-phosphate and pyruvate assay

Intracellular G6P and pyruvate levels were examined using the respective assay kits, according to the manufacturer's protocol (Sigma-Aldrich Co. LLC). In total, 2 × 10^4^ cells were either left untreated, pre-treated with inhibitors for 1 h prior to treatment with CCL5 or treated with CCL5. Bioluminescence was measured using a VICTOR™ X3 Multilabel Plate Reader (PerkinElmer).

### 4.8. Glycolytic stress test using the Seahorse Extracellular Flux Analyzer

Real-time measurements of ECAR were used as indicators of the cellular rate of glycolysis. In total, 5 × 10^4^ cells were seeded into individual wells of a 96-well plate overnight. For experiments involving inhibitors, cells were pre-treated for 1 h with the indicated inhibitor prior to experimentation. The culture medium was replaced with DMEM/F12 (without bicarbonate), to allow for a swift and rapid change in pH. The basal reading was normalized. Subsequently, compounds were injected into each individual well as indicated, to measure effects on the rate of glycolysis. Rate of change in pH was measured every 5 min. To examine glycolytic capacity, 2 µM oligomycin was injected into each well and the rapid surge and the subsequent plateau in ECAR was interpreted as the cellular transition from mitochondrial respiration to glycolysis for the production of ATP. The difference between the resulting maximum and the normalized basal ECAR was interpreted as the glycolytic capacity.

### 4.9. Proliferation assay

In total, 2 × 10^4^ cells were seeded into individual wells of 24-well plates in 2% FCS DMEM/F12. Cells were either left untreated or incubated with 10 nM CCL5 for the indicated times, then collected and counted with a haemocytometer. In CCR5 blocking studies, cells were pre-treated with inhibitors for 1 h prior to CCL5 stimulation. The medium containing CCL5 and/or inhibitors was replaced every other day.

### 4.10. Chemotaxis assay

Cell chemotaxis was assayed using 24-well Transwell chambers with 8 µm pores (Corning). In total, 1 × 10^5^ cells in 100 µl chemotaxis buffer (DMEM/F12/0.5% BSA) were placed in the upper chambers. CCL5, diluted in 600 µl chemotaxis buffer, was placed in the lower wells and the chambers were incubated for 18 h at 37°C. Cells that migrated to the bottom wells were collected and counted with a haemocytometer. For experiments involving inhibitors, cells were pre-treated for 1 h with the indicated inhibitor and then placed in the upper chambers.

### 4.11. Invasion assay

The invasive capacity of cells was assayed using a QCM ECMatrix Cell Invasion kit with 8 µm pore size, according to the manufacturer's protocol (Millipore). In total, 10^5^ cells in 100 µl buffer containing CCL5 were placed in the upper chambers. Cells were allowed 24 h to invade through the ECMatrix into the lower chamber. Cells were subsequently quantified using CyQuant GR DyeCells. Fluorimetric readings were measured using a VICTOR™ X3 Multilabel Plate Reader (PerkinElmer).

### 4.12. Metabolomic profiling

Unbiased, metabolomic profiling analysis was conducted using the Metabolon platform, as described [30]. Packed pellets of 3 × 10^7^ frozen MMTV-PyMT cells, treated as indicated, were transferred to Metabolon Inc. (Durham, NC) for metabolomics analysis. Using cold methanol extraction, supernatants from the samples were purified for mass spectrometry. To identify metabolites, the platform used ultrahigh performance liquid chromatography/tandem mass spectrometry (UHPLC/MS-MS) and gas chromatography/mass spectrometry (GC/MS) to identify features in the experimental samples against a reference library of chemical standards that include molecular weight, retention time and MS spectra. The information was then curated into standard formats for presentation purposes, including heat maps, pathways and box plot analyses, described in figures 6–8.

### 4.13. Statistical analyses

Statistical significance was analysed by Student's *t*-test unless specified otherwise. Data generated using the Metabolon platform were analysed using the Welch's *t*-test. A level of *p* < 0.05 identified significance. Data are expressed as mean ± s.e.

## Supplementary Material

CCL5 treatment activates 4E-BP1 downstream of the AKT/mTOR pathway in breast cancer cells

## Supplementary Material

MDA-MB-231, MCF-7 and MMTV-PyMT express CCR5

## Supplementary Material

CCL5 induces chemotaxis in MDA-MB-231

## Supplementary Material

Time course of glucose uptake in CCL5 treated MDA-MB-231

## Supplementary Material

CCL5 treatment results in accumulation of metabolites in MMTV-PyMT primary mouse breast cancer cells
