# Supplementary figures and images for: CCL5 activation of CCR5 regulates cell metabolism to enhance proliferation of breast cancer cells
Source: Open Biol. 2016 Jun 22;6(6):160122. doi: 10.1098/rsob.160122 (PMC4929946; doi:10.1098/rsob.160122)

Supplementary Figure S1.

A

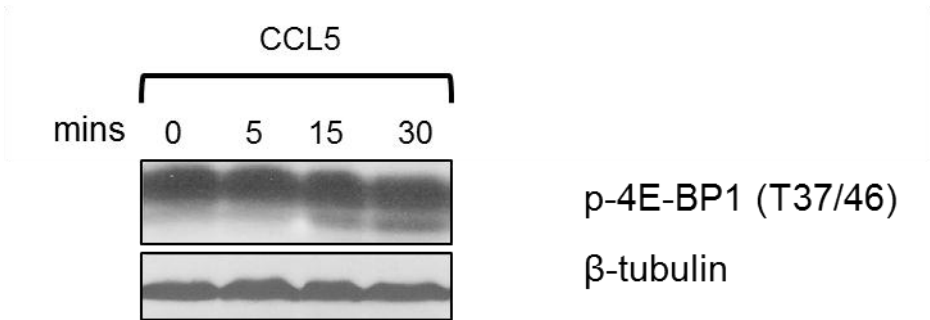

B

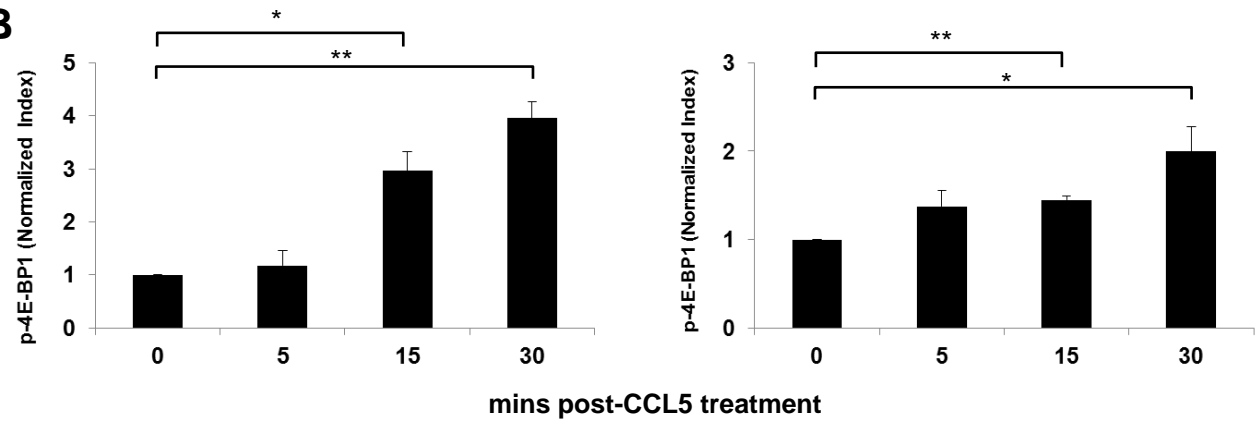

Supplement: CCL5 treatment activates 4E-BP1 downstream of the AKT/mTOR pathway in breast cancer cells [file rsob160122supp1.pdf]

Supplementary Figure S2.

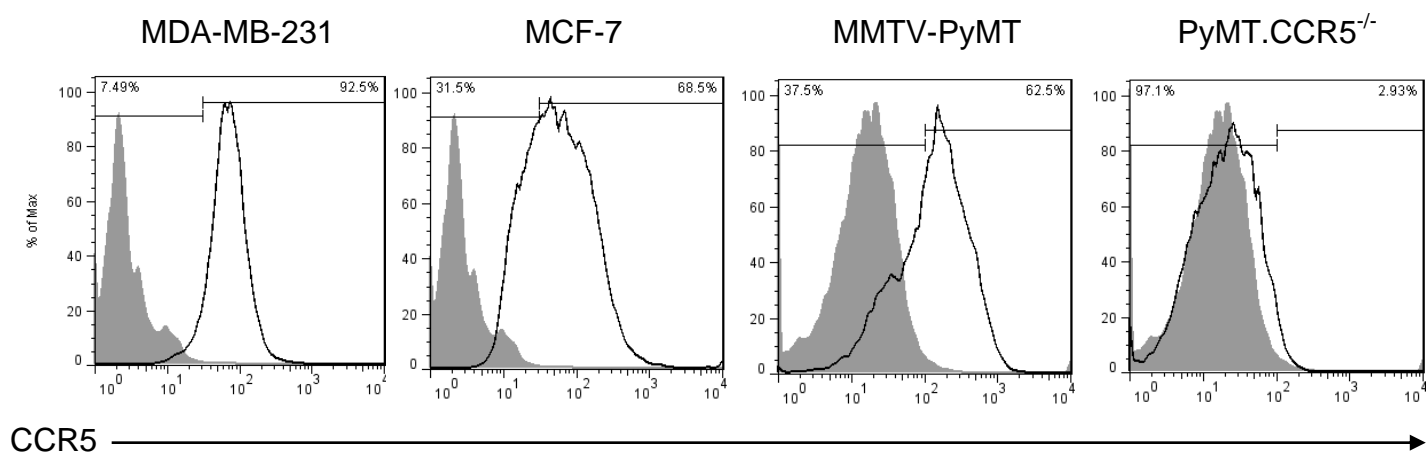

Supplement: MDA-MB-231, MCF-7 and MMTV-PyMT express CCR5 [file rsob160122supp2.pdf]

Supplementary Figure S3.

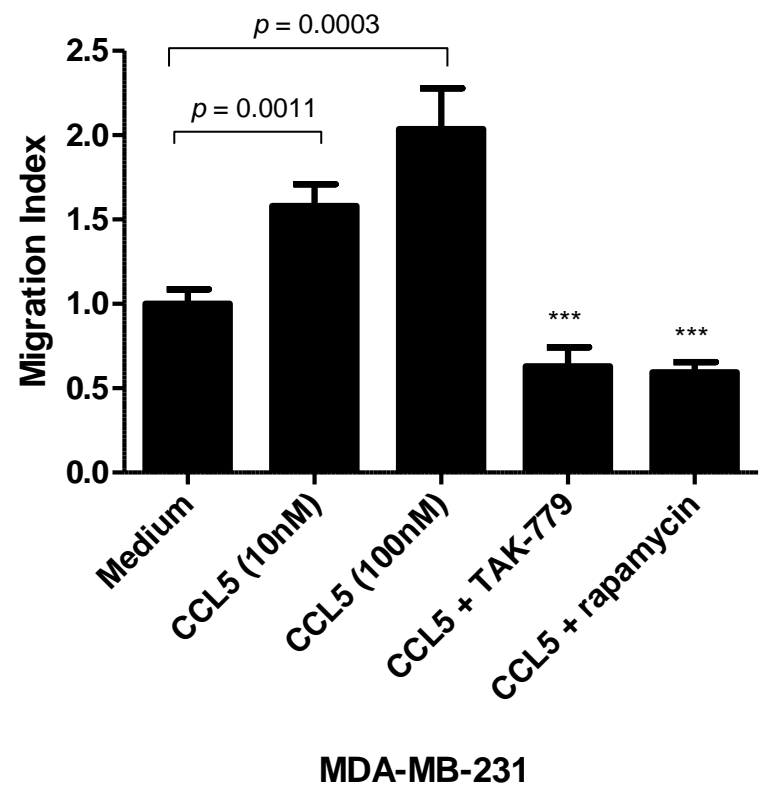

Supplement: CCL5 induces chemotaxis in MDA-MB-231 [file rsob160122supp3.pdf]

Supplementary Figure S4.

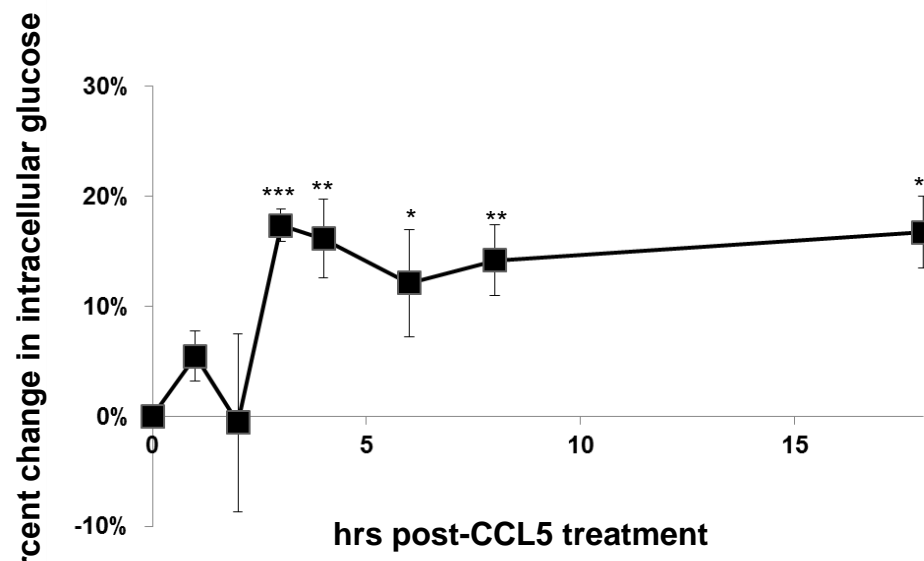

MDA-MB-231

Supplement: Time course of glucose uptake in CCL5 treated MDA-MB-231 [file rsob160122supp4.pdf]
