# Supplementary material for: CCL5 activation of CCR5 regulates cell metabolism to enhance proliferation of breast cancer cells
Source: Open Biol. 2016 Jun 22;6(6):160122. doi: 10.1098/rsob.160122 (PMC4929946; doi:10.1098/rsob.160122)
Supplement: CCL5 treatment results in accumulation of metabolites in MMTV-PyMT primary mouse breast cancer cells [file rsob160122supp5.pdf]

Supplementary Table S1.

Pathway	Metabolite	PyMT								
		Normalized Value			Fold Changes		Signficance			
		Untreated	CCL5 3h	CCL5 6h	CCL5 3h / Untreated	CCL5 6h / Untreated	CCL5 3h / Untreated		CCL5 6h / Untreated	
							p-value	q-value	p-value	q-value
Glycolysis, Gluconeogenesis, and Pyruvate Metabolism	glucose	2.4727	2.006	9.7804	0.81	3.96	0.5344	0.3366	0.0028	0.0037
	**glucose-6-phosphate (G6P)**	**0.5709**	**0.5171**	**3.7326**	**0.91**	**6.54**	**0.8198**	**0.4233**	**0.0012**	**0.0024**
	glucose 1-phosphate	1.4057	1.5452	3.1447	1.1	2.24	0.5236	0.331	0.0112	0.0079
	fructose-6-phosphate	0.6356	0.659	1.3686	1.04	2.15	0.7294	0.3969	0.0196	0.0112
	fructose 1,6-diphosphate	3.5357	5.626	12.5182	1.59	3.54	0.3063	0.2488	0.0387	0.0178
	dihydroxyacetone phosphate (DHAP)	0.5709	0.8762	2.0062	1.53	3.51	0.0554	0.1173	0.0011	0.0024
	3-phosphoglycerate	1.9383	2.1992	5.8344	1.13	3.01	0.5565	0.3463	0.0082	0.0067
	**pyruvate**	**0.8391**	**1.2159**	**1.4329**	**1.45**	**1.71**	**0.0603**	**0.1187**	**0.0114**	**0.008**
	**lactate**	**0.3171**	**0.5076**	**0.6272**	**1.6**	**1.98**	**0.0366**	**0.1032**	**0.0096**	**0.0076**
Pentose Phosphate Pathway	6-phosphogluconate	0.5529	1.1239	1.5938	**2.03**	**2.88**	0.0193	0.0946	0.0046	0.0048
	**ribose 5-phosphate**	**1.0897**	**1.2202**	**2.525**	**1.12**	**2.32**	**0.2986**	**0.247**	**0.0228**	**0.0121**
	ribose 1-phosphate	1.5153	1.6306	2.993	1.08	**1.98**	0.6573	0.3751	0.015	0.0094
	ribulose/xylulose 5-phosphate	1.4523	2.2003	3.8152	**1.52**	**2.63**	0.0361	0.1032	0.0014	0.0027
TCA Cycle	citrate	0.7289	0.3851	2.7119	0.53	**3.72**	0.5961	0.3547	0.0466	0.0203
	isocitrate	0.5044	0.0925	4.8958	**0.18**	**9.71**	0.0791	0.1288	0.016	0.0098
	**alpha-ketoglutarate**	**0.4587**	**0.4904**	**1.053**	**1.07**	**2.3**	**0.4674**	**0.3106**	**0.0002**	**0.0016**
	fumarate	0.6958	0.9764	1.4333	**1.4**	**2.06**	0.0197	0.0946	0.001	0.0024
	malate	0.9811	1.49	6.6506	1.52	**6.78**	0.3806	0.2798	0.0389	0.0178
Fatty Acid Metabolism	palmitate (16:0)	1.5697	1.9971	3.4268	1.27	**2.18**	0.1792	0.1813	0.0082	0.0067
	palmitoleate (16:1n7)	2.4005	3.5421	3.5463	**1.48**	**1.48**	0.0216	0.0946	0.0243	0.0128
	margarate (17:0)	0.757	0.9982	2.8362	1.32	**3.75**	0.1323	0.159	0.0008	0.0024
	stearate (18:0)	0.7359	1.0144	2.1393	**1.38**	**2.91**	0.0739	0.1266	0.0009	0.0024
	**stearoylcarnitine**	**0.2038**	**0.1699**	**0.6857**	**0.83**	**3.36**	**0.9298**	**0.4518**	**0.0245**	**0.0128**
	**stearamide**	**0.5817**	**0.4758**	**2.1142**	**0.82**	**3.63**	**0.4641**	**0.3106**	**0.04**	**0.018**
	**acetyl CoA**	**0.1312**	**0.3043**	**0.0574**	**2.32**	**0.44**	**0.1366**	**0.1591**	**0.5507**	**0.1392**
	**3-hydroxybutyrate (BHBA)**	**1.2586**	**3.774**	**1.146**	**3**	**0.91**	**0.0362**	**0.1032**	**0.8825**	**0.2001**
Leucine, Isoleucine and Valine Metabolism	**leucine**	**0.8326**	**0.9892**	**1.4139**	**1.19**	**1.7**	**0.2331**	**0.2083**	**0.0175**	**0.0104**
	**beta-hydroxyisovaleroylcarnitine**	**0.7348**	**0.7744**	**0.4787**	**1.05**	**0.65**	**0.6057**	**0.3566**	**0.0405**	**0.0182**
	isoleucine	0.74	0.8586	1.2463	1.16	**1.68**	0.2891	0.2405	0.0206	0.0116
	valine	0.824	0.9355	1.4115	1.14	**1.71**	0.3834	0.2798	0.0267	0.0137
Glutamate Metabolism	**glutamate**	**0.4893**	**0.513**	**1.3932**	**1.05**	**2.85**	**0.6108**	**0.3577**	**0.0008**	**0.0024**
	**glutamine**	**0.3324**	**0.3343**	**1.0822**	**1.01**	**3.26**	**0.7144**	**0.3952**	**0.0134**	**0.0089**
